# Foliar Application of Zinc Oxide Nanoparticles and Zinc Sulfate Boosts the Content of Bioactive Compounds in Habanero Peppers

**DOI:** 10.3390/plants8080254

**Published:** 2019-07-30

**Authors:** Josué I. García-López, Guillermo Niño-Medina, Emilio Olivares-Sáenz, Ricardo H. Lira-Saldivar, Enrique Díaz Barriga-Castro, Rigoberto Vázquez-Alvarado, Pablo A. Rodríguez-Salinas, Francisco Zavala-García

**Affiliations:** 1 Universidad Autónoma de Nuevo León, Laboratorio de Química y Bioquímica, Facultad de Agronomía, Francisco Villa S/N, Col. Ex-Hacienda el Canadá, C.P. 66050 General Escobedo, Nuevo León, México; 2 Departamento de Agroplasticultura, Centro de Investigación en Química Aplicada (CIQA), C.P. 25294 Saltillo, Coahuila, México

**Keywords:** *Capsicum chinense*, nanofertilizers, capsaicionoids, physicochemical quality, phenolic compounds, antioxidant capacity

## Abstract

The physiological responses of habanero pepper plants (*Capsicum chinense* Jacq.) to foliar applications of zinc sulphate and zinc nano-fertilizer were evaluated in greenhouse trials. The effect of the supplement on fruit quality of habanero pepper was particularly observed. Habanero pepper plants were grown to maturity, and during the main stages of phenological development, they were treated with foliar applications of Zn at concentrations of 1000 and 2000 mg L^−1^ in the form of zinc sulfate (ZnSO_4_) and zinc oxide nanoparticles (ZnO NPs). Additional Zn was not supplied to the control treatment plants. ZnO NPs at a concentration of 1000 mg L^−1^ positively affected plant height, stem diameter, and chlorophyll content, and increased fruit yield and biomass accumulation compared to control and ZnSO_4_ treatments. ZnO NPs at 2000 mg L^−1^ negatively affected plant growth but significantly increased fruit quality, capsaicin content by 19.3%, dihydrocapsaicin by 10.9%, and Scoville Heat Units by 16.4%. In addition, at 2000 ZnO NPs mg L^−1^ also increased content of total phenols and total flavonoids (soluble + bound) in fruits (14.50% and 26.9%, respectively), which resulted in higher antioxidant capacity in ABTS (2,2′azino-bis(3-ethylbenzothiazoline-6-sulfonic acid)), DPPH (2,2-diphenyl-1-picrylhydrazyl), and FRAP (ferric reducing antioxidant power) (15.4%, 31.8%, and 20.5%, respectively). These results indicate that application of ZnO NPs could be employed in habanero pepper production to improve yield, quality, and nutraceutical properties of fruits.

## 1. Introduction

The addition of fertilizers to supplement the natural fertility of the soil is essential for modern crop production, and precise nutrient management is indispensable for sustainable agricultural production [1]. Zinc (Zn) plays an important role in plant functions, it modifies auxin effects through regulation of tryptophan synthesis, and it acts as a cofactor in the redox enzymes like superoxide dismutase and dehydrogenases [2].

Micro-elements play important roles in plant development, fruit yield and quality [3,4]. Zinc is an essential micro-nutrient for humans, animals, and plants. Plants generally absorb Zn as a divalent cation (Zn^++^). Zinc is required in protein biosynthesis and carbohydrate metabolism, and plays an important role in gene expression related to environmental stress [5,6]. Although Zn is required by plants for optimal metabolism, the efficiency of this micro-element depends on its absorption and translocation [7]. Traditional agriculture practices employ Zn sulphate (ZnSO_4_) or EDTA-Zn chelate for application to leaves and ground. However, the efficiency is low [8]. Zn absorption through the leaves seems to be determined by the source of the micro-nutrients. For example, in a study carried out by Doolette et al. [9] on plants of *Triticum aestivum* cv Shield, when comparing foliar fertilization with ZnSO_4_ and ZnEDTA at a concentration of 1000 mg L^−1^, there was a significantly higher proportion of Zn on the leaves treated with ZnSO_4_ compared to leaves treated with ZnEDTA. The foliar application of Zn is an effective way to boosts the absorption of Zn in plants, however, the development of Zn-based foliar fertilizers is compromised by the lack of knowledge, development of new materials, mobility, and Zn speciation within the plant [9,10]. In Mexico, the habanero pepper is consumed mainly in the states of Yucatan, Quintana Roo, Campeche, and Tabasco, with a growing demand in the Mexican and international markets. However, current crop yields do not meet local demand, mainly due to limited technology in their production and adequate supply of fertilizers through the irrigation system [11]. Therefore, an optimized fertilization method that increases crop yield and reduces pollution by using more efficient fertilizers is needed. The use of Zn-based foliar fertilizers during the development of the crops can be an effective way to increase the assimilation of Zn and increase the yield, however, the soluble salts of Zn can cause damage to the leaf and its cost is very elevated [10]. While, the ZnO NPs are considered a biosecure material for biological species, since their efficiency has been demonstrated to promote the germination of seeds and the growth of plants, as well as in the suppression of disease and the protection of plants for their antimicrobial activity [12]. The concentration of Zn in the soil solution is very low and occurs in the form of various types of salts including ZnS, sphalerite (ZnFe)S, ZnO zincite and smithsonite ZnCO_3_, however, the absorption of this element by plants is determined by the concentration of carbonates (CaCO_3_) and soil pH, which are the main causes that limit the availability of this micronutrient [13]. As a consequence, there is a growing interest in foliar fertilization for sustainable crop management. Foliar fertilization solves limited nutrient availability by minimizing losses of fertilizer applied to the soil and that limit the delivery of nutrients to the organs of the plant during critical stages of growth [14].

Nanotechnology, with the use of nanoparticles (NPs), is providing novel approaches to plant nutrition [15]. Fertilizers at the nanometer scale (1–100 nm) increase greatly the points of impact because of their reduced size, which in turn could improve the interaction and uptake of micronutrients in crop fertilization [12]. Foliar applications of nano-fertilizers have proven to be effective because they supply nutrients to plants in a gradual and controlled manner compared to conventional fertilization [16,17]. Application of nano-fertilizers also requires smaller quantities than conventional fertilizers [1]. A study conducted by Rossi et al. [18] showed that foliar application of ZnO NPs positively influenced growth and physiology of coffee plants (*Coffea arabica* L.), even more than Zn (ZnSO_4_) salts application, due to greater leaf penetration. Research using pomegranate trees (*Punica granatum* cv. Ardestani) showed that foliar fertilization with relatively low amounts of Zn and B nano-fertilizers modified yield and fruit quality, and juice sugars and maturity index increased [1]. Our research group has also observed positive effects of ZnO NPs on *Capsicum chinense* germination, that, in turn, positively affected physiological variables (seed germination, seedling vigor, and biomass accumulation) and nutraceutical properties (total phenols, total flavonoids, condensed tannins, and DPPH antioxidant capacity) [19].

The effects of ZnO NPs on plants result from changes in the physical, chemical, and biological characteristics of the materials applied as nano-fertilizers, as well as on their catalytic properties. These changes consequently affect chemical and biological activities that could induce oxidative stress and toxicity in plants and stimulate the antioxidant systems [20,21]. A recent study concluded that nano-toxicity depends on the composition and concentration of the NPs and the species evaluated [22].

Although, the effect of ZnO NPs has been reported in crops like onion (*Allium cepa* L.), green pea (*Pisum sativum* L.) and spinach (*Spinacia oleracea*) [23,24,25], no studies have been published describing the interaction of ZnO NPs and ZnSO_4_ in the habanero pepper. Thus, this research compared growth responses of pepper plants to foliar applications of zinc sulphate (ZnSO_4_) and zinc nano-fertilizer (ZnO NPs), and analyzed the quality and accumulation of bioactive compounds of pepper fruits obtained from plants treated with ZnSO_4_ and ZnO NPs to understand their effect on the physiology of pepper plants.

## 2. Materials and Methods

### 2.1. Plant Material

The *Capsicum chinense* variety used was Chichen Itza (Seminis, St. Louis, MO, USA, EE.UU.). It is a vigorous and early maturing plant that can be harvested up to two weeks earlier than other varieties. Fruits have three lobes and an attractive orange color. The fruits are characteristically spicy, 10,500 SHU units fresh and up to 200,000 SHU dry.

### 2.2. Characteristics of the ZnO NPs Used in This Experiment

The morphological and structural characterization of the ZnO NPs used in this study has been reported previously by García-López et al. [26]. Specifically, most of the particles (75%) had diameters of 12 to 24 nm, and 30% showed sizes greater than 12 nm and smaller than 20 nm.

### 2.3. Preparation of Suspensions

ZnSO_4_ and ZnO NPs suspensions were prepared just prior to exposure in deionized water (DI) and homogenized with a probe sonicator (Qsonica, CT, USA) for 30 min at 120 volts, 3 amps and 50 to 60 GHz. Subsequently, pH for each suspension was adjusted to 6.5–6.7 with HCl and 0.1 N NaOH before exposure to the plant [27]. The treatments applied were: absolute control without Zn, 1000 mg L^−1^ and 2000 mg L^−1^ with ZnSO_4_, 1000 mg L^−1^ and 2000 mg L^−1^ with ZnO NPs, with five repetitions for each treatment. The experimental unit was one plant per pot. The concentrations used were selected from previous studies conducted by Torabian et al. [28] and Kisan et al. [29]. Electrical conductivities (EC) of the suspensions with ZnSO_4_ were 0.97 and 1.64 mS cm^−1^ for 1000 and 2000 mg L^−1^, while the CE of the suspensions with ZnO NPs were 0.68 and 1.11 mS cm^−1^ for 1000 and 2000 mg L^−1^, respectively. The plants of the control treatment were treated with distilled water with an EC of 0.37 mS cm^−1^.

### 2.4. Foliar Exposure to Zn

**Figure 1:**
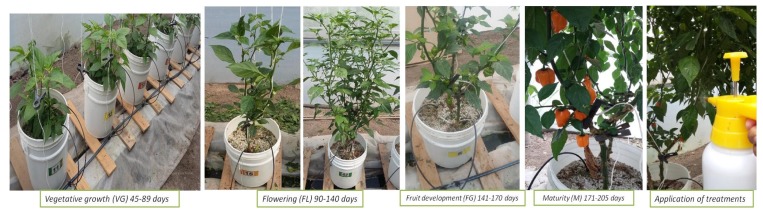
Schematic representation of each of the vegetative stages of the crop, additionally, a representative image of the way in which the treatments were applied is presented.

Approximately 0.125 L of the Zn suspensions was sprayed to cover the foliage twice in each of the following stages of crop development: vegetative growth (VG) 45–89 days, flowering (FL) 90–140 days, fruit development (FG) 141–170 days, and maturity (M) 171–205 days, to give a total amount of 0.8 and 1.6 mg of zinc per plant. For foliar application, a 1.2 L hand Hudson RL Flomaster sprayer (Lowell, MI, USA) was used, it was equipped with a brass nozzle and a safety valve. Crop conditions are explained in the following section. In Figure 1, each of the phenological stages of the crop is shown in a representative manner, in addition to the foliar application of the treatments.

### 2.5. Growth of Habanero Pepper Plants and Greenhouse Conditions

Habanero pepper plants were established in an Israeli-type greenhouse equipped with semi-automatic side windows and zeniths covered with polyethylene. The experiment was carried out for 208 days after transplant. Average temperature was 22.1 °C at a relative humidity of 79%. Planting density was 2.4 plants per m^2^. Pumicite was used as a growth medium in 19-L white plastic containers.

An automated drip-irrigation system watered eight times per day for a total of approximately 3.2 L of water per plant. The nutrient solution was based on Steiner [30] at pH of 6.5. During vegetative growth, the concentration was 25%, and it increased to 50% during flowering and fruit development [31]. Each pot was provided with equal amounts of nutrient solution. The nutrient solution did not contain Zn.

### 2.6. Performance Variables and Relative Chlorophyll Content

During the plant life cycle, physiological parameters such as plant height, stem diameter, and chlorophyll content were measured at 45, 90, 145, and 201 days. These dates coincide with vegetative growth, flowering, fruit development, and maturity, respectively. Relative chlorophyll content was determined in fresh plants, with a portable chlorophyll meter (SPAD-502, Minolta, Osaka, Japan). Data reported are averages of three measurements per leaf for each experimental unit [32].

At the end of the crop, the numbers of fruits harvested per plant, the average weight of fruits (g), the yield per plant (total weight of fruits g), and the fresh and dry weight of aerial biomass (g) were quantified. This data set was registered at 208 days to evaluate the growth and performance of habanero pepper plants. The dry weight of aerial biomass (g) was obtained after drying in a Lab-Line stove (Model 3478M, Iztapalapa, Mexico) at a constant temperature of 70 °C for a period of 72 h.

### 2.7. Preparation for the Analysis of Habanero Pepper Fruits

In this study, fruits were harvested 208 days after sowing, in the fully mature stage (orange color). Afterwards, they were washed with a 3% sodium hypochlorite solution and dried in a cool and ventilated place [33]. After drying, the effect of ZnSO_4_ and ZnO NPs on fruit quality was evaluated on 10 uniform fruits without defects (physical or pathological damage) selected from each of the five replications per treatment [34]. A total of 50 fruits per treatment were sampled: 25 fruits were used for physical and chemical analysis, and the remaining 25 fruits were for functional analyses [35].

#### 2.7.1. Physicochemical Fruit Evaluations

Chromatic evaluations were carried out on the fruit bark with a CR-10 Konica Minolta colorimeter (Tokyo, Japan). Chromatic parameters were obtained using the CIELAB (L*, a*, b*) and CIELCH (L*, C*, *h*) color systems according to the Commission Internationale De L’ecleirage (CIE) [36]. L* defines luminosity (0 black, 100 white), a* indicates red (positive a*) or green (negative a*), b* indicates yellow (positive b*) or blue (negative b*), C* (chrome, saturation level of *h*) and *h* (tone angle: 0° = red, 90° = yellow, 180° = green, 270° = blue). Color display was obtained using the ColorHexa [37] online software using the L*, a* and b* values.

Firmness and cutting force for the fruits was evaluated with an Ametek Chatillon DFS II strength meter adapted to a manual rotating system (West, Sussex, UK). Fruits were deformed a total distance of 2 mm with a 30 mm compression plate, the maximum force required was recorded in Newton (N). After physical evaluations, fruits were stored at −20 °C until chemical evaluations were performed.

The total soluble solids were determined by manually squeezing the juice of each fruit in a refractometer (Atago MASTER-M, Tokyo, Japan) with a scale of 0 to 33%. For the determination of titratable acidity (TA) and pH, fruits were cut into slices with a knife and placed inside a polyethylene bag to produce a homogeneous mixture. Then 5 g of sample were taken (pericarp and placenta without seed), 25 mL of water added, and the mixture processed for 40 s in an Oster blender (M4655-813/465-42, Sunbeam, Mexico City, Mexico). The extract was filtered through organza fabric. The pH was measured using a bench-top meter (pH/ORP Meter HI 2221, Cd de México, México). Titratable acidity (TA) was determined using the Association of Official Analytical Chemist (AOAC) [38] method 942.15 with 0.1 M NaOH at pH 8.2 using phenolphthalein as indicator. Titratable acidity was reported in % of citric acid in fresh weight.

#### 2.7.2. Sample Preparation for Capsaicinoids, Phenolics and Antioxidant Capacity Analysis

Habanero pepper fruits were sliced with a knife, and the seeds were removed to determine bioactive and functional compounds. Subsequently, fruits were placed in a polyethylene bag to make a homogeneous mixture, and samples were stored in an ultra-freezer (3003 Ultrafreezer Thermo Scientific, Waltham, MA, USA) at −80 °C until analysis.

#### 2.7.3. Capsaicinoids Extraction

Extraction and quantification of capsaicinoids were based on the method established by Ryu et al. [39]. Five grams of fresh pepper sample (pericarp and placenta without seed) were weighted, and 25 mL of acetone was added. The mixture was processed for 30 s in an Oster blender (M4655-813/465-42, Sunbeam, Mexico City, Mexico). The extract was heated to 50 °C for one hour in a laboratory oven (ON-12G, Jeio Tech, Seoul, Korea). After this period, samples were centrifuged at 4500× *g* for 5 min, and the supernatant was recovered for analysis.

#### 2.7.4. Quantification of Capsaicin and Dihydrocapsaicin by HPLC

For HPLC analysis, extracts with acetone were filtered with a 0.25 mm syringe filter with a pore size of 45 μm, and 10 μL were injected directly into the system Agilent Technologies 1260 Infinity HPLC, equipped with a 1260 G1311C quaternary pump, a G1316A thermostatted column compartment, a G1329B autosampler and a G4212B diode array detector (Santa Clara, CA, USA). The column used was a ZORBAX Eclipse Plus C-18 analytical column (100 mm × 3 mm id, 5 μm). The isocratic mobile phase included 100% acetonitrile (solvent A) and 1% acetic acid in water (solvent B) at a 40:60 ratio and a flow rate of 1 mL min^−1^ at 25 °C, with a run time of 20 min. Absorbance was measured at 280 nm.

The quantification of capsaicin (8-methyl-*N*-vanillil-6-nonenamide) and dihydrocapsaicin (8-methyl-*N*-vanillilnonamide) (Sigma Aldrich, St. Louis, MO, USA) was based on corresponding calibration curves at concentrations of 0, 80 mg kg^−1^, 160 mg kg^−1^, 240 mg kg^−1^, 320 mg kg^−1^ [39].

#### 2.7.5. Scoville Heat Units Calculation

Capsaicinoid content (mg kg^−1^) was transformed into Scoville Heat Units (SHU) as established by Todd et al. [40]. Determinations of the pungency in SHU were made by multiplying the individual contents of capsaicinoids (capsaicin and dihydrocapsaicin) by the value corresponding to the intensity of the threshold.

[(% Capsaicin × 16.1) + (% Dihydrocapsaicin × 16.1)] × 10,000 = SHU
(1)

### 2.8. Extraction of Soluble and Bound Phenolic Compounds

Soluble extracts were obtained by suspending 5 g of the fresh pepper sample (pericarp and placenta without seed) in 50 mL of 80% methanol. The sample was purged with an argon stream for 30 s and mixed for 45 s in an Oster blender (M4655-813/465-42, Sunbeam, Mexico City, Mexico). Finally, the mixture was filtered with organza fabric to separate the insoluble matter from the juice, and it was placed in 15 mL centrifuge tubes. The extract was centrifuged at 4500× *g*, and the supernatant was recovered and stored at −20 °C until analysis.

Bound phenolics were extracted by suspending the remaining insoluble matter in 10 mL of 2 mol L^−1^ NaOH and purged with argon for 30 s. The mixture was then shaken at 200 rpm for 2 h. After that, the pH was adjusted to 2.5 with concentrated HCl and centrifuged at 4500× *g*. The supernatant was recovered, and the phenolic compounds were extracted with 10 mL of ethyl acetate twice. The ethyl acetate extracts were combined and evaporated at 40 °C with argon. The dried extract was stored at −20 °C, and before analysis, it was suspended in 3 mL of 80% methanol. The extracts obtained from soluble and bound phenolic compounds were used for the trials of total phenols, total flavonoids, and antioxidant capacity.

#### 2.8.1. Determination of Total Phenols

Phenolics compounds and antioxidant capacity assays were performed in a Barnstead International Turner SP-830 Plus spectrophotometer (Dubuque, IA, USA). To determine the content of phenols, 0.2 mL of each extract were taken and mixed with 2.6 mL of distilled water and 0.2 mL of Folin-Ciocalteu reagent. After 5 min, 2 mL of 7% Na_2_CO_3_ was added, and the solution was stirred for 30 s. The reaction took place in the dark for 90 min, after which the absorbance of the samples was measured at 750 nm. The concentration of phenols was reported in equivalent milligrams of gallic acid per kilogram of the sample (mgGAE kg^−1^), calculated from the calibration curve of gallic acid from 0 to 200 mg L^−1^. 

#### 2.8.2. Determination of Total Flavonoids

Flavonoid content was determined by the reaction of the AICI_3_-NaNO_2_-NaOH complex. From the extract, 0.2 mL were mixed with 3.5 mL of distilled water. Subsequently, 0.15 mL of 5% NaNO_2_, 0.15 mL of 10% aluminium chlorhidre (AlCl_3_) and 1 mL of 1 M NaOH were added at 5 min intervals between each reagent. The reaction proceeded for 15 min, and then the absorbance at 510 nm was measured. Total flavonoid content was reported in milligrams of equivalent catechin per kilogram of the sample (mgCatE kg^−1^), calculated from the catechin calibration curve from 0 to 200 mg L^−1^.

#### 2.8.3. Determination of Condensed Tannins

Condensed tannins content was determined by the reaction of the vanillin-H_2_SO_4_ complex. From the extract, 0.25 mL was taken and mixed with 0.65 mL of 1% vanillin, then, 0.65 mL of 25% H_2_SO_4_ was added. Vanillin and H_2_SO_4_ were dissolved in methanol. The reaction proceeded for 15 min at 30 °C, and then absorbance at 500 nm was measured. Condensed tannins content was reported in milligrams of equivalent catechin per kilogram of the sample (mgCatE kg^−1^), calculated from the calibration curve for catechin from 0 to 200 mg L^−1^.

#### 2.8.4. Antioxidant Capacity

The antioxidant capacity of DPPH (2,2-diphenyl-1-picrylhydrazyl) was evaluated using a 60 μM working solution in 80% methanol, with an absorbance adjusted to 0.98 at 517 nm, with 0.055 error. The assay was carried out by mixing 50 μL of the extract with 1.5 mL of the DPPH working solution; the reaction was left for 30 min in the dark, and the absorbance was determined.

The antioxidant capacity of ABTS (2,2′azino-bis(3-ethylbenzothiazoline-6-sulfonic acid)) was determined using a working solution obtained by mixing 1 mL of ABTS at 7.4 mM and 1 mL of K_2_S_2_O_8_, and allowing them to react for 12 h in the dark. The absorbance of the working solution was adjusted to 0.97 to 734 nm by diluting with methanol. The ABTS assay was performed by mixing 50 μL of the extract with 1.5 mL of ABTS working solution. The reaction was left for 30 min in the dark, and the absorbance was measured.

The antioxidant capacity of FRAP (ferric reducing antioxidant power) was determined using a working solution prepared by mixing 300 mM C_2_H_3_NaO_2_·3H_2_O (pH 3.6), 10 mM ·TPTZ (2,4,6-tripyridyl-s-triazine, in 40 mM HCl), and 20 mM FeCl_3_·6H_2_O in a 10:1:1 ratio (V:V:V). The FRAP assay was prepared by mixing 50 μL of the extract with 1.5 mL of FRAP working solution; the reaction was left for 30 min in the dark at 37 °C, and the absorbance of the samples was taken at 593 nm.

Antioxidant capacity for the DPPH, ABTS, and FRAP assays were reported in millimoles of equivalent Trolox (6-hydroxy-2,5,7,8-tetramethylchroman-2-carboxylic acid) per kilogram of the sample (mmoL TE kg^−1^), according to the calibration curve with Trolox in concentrations from 0 to 500 mmoL L^−1^.

The phenolic compound and the antioxidant capacity evaluations were done according to López-Contreras et al. [41].

### 2.9. Experimental design and Statistical Analysis

The crop was established using a completely randomized experimental design, with five treatments and five experimental units per treatment. Information from developmental variables for the crop (plant height, stem diameter, and chlorophyll content) was analyzed with a factorial arrangement of 5 × 4 (A × B). Factor A was the ZnSO_4_ and ZnO NPs concentrations (control, 1000 and 2000 mg L^−1^), and factor B was the phenological stages of the crop (vegetative growth, flowering, fruit development, and maturity). Each plant was considered an experimental unit.

For the performance variables and physicochemical analysis, a completely randomized design with five experimental units per treatment was used, except for the functional analysis of capsaicinoids, phenolic compounds, and antioxidant capacity which included three experimental units. Results were reported as mean ± standard deviation. Statistically significant differences between samples were analyzed with ANOVA, and the treatment means were compared with a Tukey test (*p* ≤ 0.05) using the statistical package SPSS version 21.0 (SPSS Inc., Chicago, IL, USA).

## 3. Results and Discussion

### 3.1. Growth of Pepper Plants

**Figure 2:**
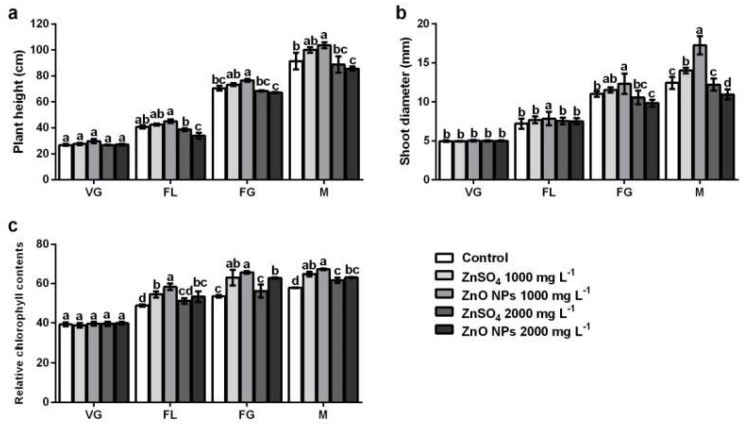
Effect of foliar application of ZnSO_4_ and ZnO NPs on agronomic parameters (**a**) plant height (**b**) shoot diameter, (**c**) relative chlorophyll content characteristics in different phenological stages of the plant. (VG) vegetative growth, (FL) flowering, (FG) fruit development, (M) maturity. The values are the average of five repetitions. Means (*n* = 5). Bars represent the standard deviation of the mean. Different letters mean that treatments were statistically diferentes (Tukey, *p* ≤ 0.05) and represent the differences between treatments, whitin each phenological stage.

The variables of agronomic behavior were evaluated during the phenological development of the plants. The growth of the plants increased significantly from the flowering stage with the foliar application of ZnO NPs at a concentration of 1000 mg L^−1^, but no significant differences were found between ZnSO_4_ and the control (Figure 2a). However, it is important to note that the greatest increments in plant height were obtained with application of ZnO NPs at 1000 mg L^−1^. This behavior is enhanced from the FL, FG, and M stages, with increases of 10.6%, 8.6%, and 13.4%, respectively, compared to the control treatment. When compared with plants treated with ZnSO_4_, although no significant differences were observed, increments of 5.8%, 4.2%, and 3.6%, respectively, were obtained. On the other hand, application of ZnO NPs and ZnSO_4_ at 2000 mg L^−1^ negatively affected plant height. Reductions with ZnO NPs were 16.7%, 4.7%, and 6.2%, while ZnSO_4_ treatment reduced plant height by 5.1%, 3.0%, and 2.9%, in comparison to the control treatment.

The behavior in the development of stem diameter (Figure 2b) was similar to that of plant height, from stages FL, FG, and M, ZnO NPs at 1000 mg L^−1^ promoted stem growth. It should be noted that the increases obtained were 8.3%, 10.7%, and 27.9% higher than the control treatment, whereas, when compared to plants treated with ZnSO_4_, the increases were 2.1%, 6.5%, and 18.7%, respectively. However, at 2000 mg L^−1^ of ZnO NPs and ZnSO_4_, height reductions were observed from stage FG to M. In the treatment with ZnO NPs at 2000 mg L^−1^, reductions were 10.5% and 11.6%, while ZnSO_4_ treatment reduced height by 4.1% and 1.5% compared to the control treatment plants.

Results from this research suggest that foliar application of ZnO NPs at 1000 mg L^−1^ had a greater impact on plant growth and physiology than conventional Zn (ZnSO_4_) salts, probably due to greater capacity to be absorbed by the blade. Rossi et al. [18] compared the foliar fertilization of ZnO NPs and ZnSO_4_ in plants of (*Coffea arabica* L.) at 10 mg L^−1^ concentrations, and concluded that leaves treated with ZnO NPs contained higher Zn concentrations (1267.1 ± 367.2 mg kg^−1^ DW) compared to plants treated with ZnSO_4_ (344.1 ± 106.2 mg kg^−1^ DW), while control plants had only a small amount of Zn in their leaves (53.6 ± 18.9 mg kg^−1^ DW). This accumulation resulted in positive effects on the development of leaves and roots by 95% and 37%, respectively, compared to the control treatment. Altogether, these results indicate that Zn assimilation is more efficient when using particles of nanometric size. Similar findings were found by Pavani et al. [42]. They showed an increased growth in seedlings treated with ZnO NPs, while seedlings grown in ZnSO_4_ grew slower. Foliar nanofertilizers can be more effective than conventional foliar fertilizers, since their release can be slow and gradual [43], but it is not yet specified if this effect is due to the absorption of the nanoparticle or due to the dissolution of its products [44,45].

However, at concentrations of 2000 mg L^−1^ affected the development of the plant, possibly due to a phytotoxic effect. The difference in physiological impact between ZnO NPs and ZnSO_4_ is attributed to a slower and more gradual release of the Zn^++^ contained in the ZnO NPs. A study conducted by Reed et al. [46] mentioned that dissolution of ZnO NPs in deionized water (DI) is relatively slow and gradual, with only 2% of dissolved Zn starting after 24 h. On the contrary, ZnSO_4_ is highly soluble with very little retention within the plant. Therefore, Zn bioavailability over a prolonged period is inefficient [16].

### 3.2. Relative Chlorophyll Content

Relative chlorophyll content was determined by SPAD measurements during the phenological development of the plants. As shown in Figure 2c, results indicate that relative chlorophyll content increased significantly during the development of the plants and by foliar application of ZnO NPs and ZnSO_4_ at both concentrations. However, leaves that accumulated the highest chlorophyll content were treated with ZnO NPs at 1000 mg L^−1^. This behavior was observed from stages FL, FG, and M, with rises of 19.4%, 22.9%, and 16.2%, respectively, compared to the control. In contrast, foliar applications for both treatments at 2000 mg L^−1^ showed reductions: with ZnO NPs, chlorophyll content decreased 8.5%, 4.3%, and 6.2%, while ZnSO_4_ caused reductions by 12.2%, 14.4%, and 8.4% in stages FL, FG, and M, respectively, compared to the treatment that produced the highest accumulation (ZnO NPs at 1000 mg L^−1^).

Our results are consistent with Prasad et al. [16], who reported a higher content of chlorophyll (1.97 mg g^−1^ rt.wt) in peanut leaves by foliar application of ZnO NPs at 1000 mg L^−1^ (25 nm) compared to ZnSO_4_. Mukherjee et al. [24], in studies conducted in green pea plants (*Pisum sativum* L.), evaluated the impact of different ZnO NPs (10 nm), 2% by weight of doped alumina (Al_2_O_3_ ZnO NPs, 15 nm), 1% by weight of NPs coated with aminopropyltriethoxysilane (KH550, ZnO NPs 20 nm) and ionic Zn (Zn chloride) at 1000 mg kg^−1^. All treatments resulted in 2.4 to 3.6 fold increments in chlorophyll accumulation, compared to the control, although there were no significant differences between different Zn types.

The observed increases in the chlorophyll content are due to the fact that Zn plays an essential role in the metabolism of plants, by influencing the activity of important enzymes such as carbonic anhydrase containing a Zn atom that catalyzes the hydration of CO_2_ that facilitates the diffusion of carbon dioxide to the carboxylation sites in plants [47,48]. The above is in agreement with the findings reported by Pullagurala et al. [48], indicated that the application ZnO NPs at 100 mg kg^−1^, 200 mg kg^−1^, and 400 mg kg^−1^ increased the relative chlorophyll content by 41%, 37%, and 58%, respectively, in comparison with the control plants. According to Raliya and Tarafdar [27], the foliar application of ZnO NPs to plants of *Cyamopsis tetragonoloba* L. at 10 mg L^−1^ significantly increased the biomass of the plant (27.1%) and the chlorophyll content (276.2%). Thus, the observed increases in plant growth at a concentration of 1000 mg L^−1^ with ZnO NPs are due to the increase in chlorophyll content, since it is a common indicator of the photosynthetic efficiency of a plant, which is one of the most important determinants of its growth [21].

Even though Zn is an essential micronutrient for growth and metabolism of plants [2] and it is necessary for chlorophyll production [49], harmful responses to high Zn concentrations may be closely related to the generation of reactive oxygen species (ROS). Additionally, displacement of some other metals from the active sites in proteins [50] can affect chlorophyll biosynthesis and damage the photosynthetic system [51]. Chlorophyll content has been classified as a reliable indicator of contamination and toxicity of heavy metals in plants [52]. Therefore, high levels of Zn in plants could reduce chlorophyll content as a response to oxidative stress by this element.

### 3.3. Fruit Yield and Plant Biomass

Results for variables associated to yield and biomass accumulation are shown in Table 1. For all the variables tested, significant differences were obtained (*p* ≤ 0.01). Plants exposed to foliar application of 1000 mg L^−1^ of ZnO NPs produced the highest number of fruits, exceeding by 15.3% and 8.6% the control treatment and the ZnSO_4_-treated plants. In contrast, at 2000 mg L^−1^ ZnO NPs and ZnSO_4_, there were slight decreases (13.6% and 7.3%), compared to the control treatment. Similarly, maximum average fruit weight was obtained with ZnO NPs at 1000 mg L^−1^, exceeding by 7% the control treatment and by 3.6% the ZnSO_4_ treatment. At 2000 mg L^−1^ of ZnO NPs and ZnSO_4_, there are reductions of 5.8% and 3.8% respectively, compared to the control treatment. The trend was similar for fruit total weight: the highest weight was obtained with ZnO NPs at 1000 mg L^−1^ (Table 1), exceeding by 21.2% the control and 11.8% the ZnSO_4_ treatment. At 2000 mg L^−1^, both treatments (ZnO NPs and ZnSO_4_) showed decreases of 18.6% and 10.8%, respectively, in comparison to the control.

The dry and fresh weight of aerial biomass was significantly affected (*p* ≤ 0.01) by the foliar application of ZnO NPs and ZnSO_4_. Plants treated with 1000 mg L^−1^ of ZnO NPs showed the greatest increase in biomass accumulation: in the case of fresh weight, it increased by 3.4% and 2.2%, while dry weight increased by 9.2% and 4.3% compared to control and ZnSO_4_ treatments. However, at 2000 mg L^−1^ of ZnO NPs, the greatest reductions in fresh and dry biomass accumulation were obtained: reductions of 2.9% and 10.3% respectively, compared to the control.

In this research, foliar application of ZnO NPs at 1000 mg L^−1^ positively influenced growth of pepper plants and fruit production, and it produced more favorable effects than ZnSO_4_ fertilization. This could be due to greater Zn assimilation when applied in the form of ZnO NPs with greater leaf penetration ability [18], however, higher concentrations (2000 mg L^−1^) caused toxicity. These results agree with those reported by Lin and Xing [53] in radish, rapeseed, corn, lettuce, and cucumber. Prasad et al. [16] mentioned that ZnO NPs are absorbed by plants to a greater extent compared to ZnSO_4_ because of greater bioavailability due to their size and lower solubility in water. They tested this behavior in peanut plants. Application of 1000 mg L^−1^ of ZnO NPs increased more effective growth and yield of the pod by 34% compared to ZnSO_4_ treatment, however, at a higher concentration (2000 mg L^−1^) ZnO NPs were harmful. Similar results were obtained by Khanm et al. [54] when treating tomato plants with 1000 mg L^−1^ of ZnO NPs. They observed that plant growth, yield, and Zn accumulation increased significantly with respect to ZnSO_4_ treatment and control. These results confirmed that the physiological effects were related to greater availability of Zn when applied with particles of nanometric size.

The effectiveness of ZnSO_4_ by the foliar application is low because it is highly soluble and leaches quickly [8]. These characteristics affect nutrient availability. Highly water-soluble ions may have difficulty penetrating the lipophilic cuticle, thus limiting availability in the case of ZnSO_4_ fertilization [16]. Yet, NPs have been shown to enter cells through the stomatal or vascular system [27,55] depending on the size range of ZnO particles. This supports the current hypothesis of NPs penetration in the plant cell through the hydrophilic pathway of the polar aqueous pores in the cuticle and stomata [56,57]. The diameter of cuticular pores has been estimated at 2 nm [56], and the stomatal path appears as the most feasible route for the penetration of NPs, with a limit of size exclusion above 10 nm [57]. However, there are some alternative findings in the literature, which reported that the foliar application of Au NPs at 280 ng per plant in sizes of 3, 10, and 50 nm, managed to penetrate the leaf cuticle independently of its size and coating and, they accumulated mainly in younger shoots (10–30%) and in the roots (10–25%), and 5–15% of the NPs < 50 nm were exuded to the rhizosphere soil [58]. Other authors mention that NPs in a range of 4 to 100 nm can cross the cuticle of the leaf by breaking the waxy layer [59]. In this study, the applied nanoparticles were in a range of 12 to 24 nm, so there is the possibility that the largest size ZnO NPs (24 nm) could pass through the cuticle of the leaf. Although, it is probable that the size and properties of NPs play an important role in their interaction with the leaves of the plant, their absorption in the leaf and their transport within the plant is determined by the different characteristics of the leaf (such as the stomata, trichomes, or cuticle), which present a wide range of diversity among species [58,60].

On the contrary, reduction in biomass accumulation could be due to a greater negative impact of ZnO NPs, toxic Zn concentrations (2000 mg L^−1^) could negatively affect cellular K^+^ content, permeability, hydraulic conductivity, and water content, which decrease fresh weight of the organs, assimilate movement from leaves to fruits, and yield [61].

### 3.4. Chromatic Characteristics

Determination of chromatic characteristics of the fruits revealed significant differences (*p* ≤ 0.01) among treatments for variables L*, b*, and C* (Table 2). Although in a* and *h* the treatments were statistically equal, values showed a tendency to increase with applications of ZnO NPs and ZnSO_4_, but the differences were not significant. Maximum values of L* (53.46) were detected in the fruits from plants treated with ZnO NPs at 2000 mg L^−1^, which indicates greater luminosity of the fruits when compared to values obtained in the control and ZnSO_4_ treatments. Readings for a* and b* showed the same tendency, with values of 33.45 (a*) and 43.59 (b*). This indicates that the tendency to the red and yellow color of the fruit increased with the application of ZnO NPs at high concentrations (2000 mg L^−1^).

Color saturation (C*) presented a pattern similar to that of L* with a value of 53.43. This means that the intensity of the orange color of the fruit increased with ZnO NPs at 2000 mg L^−1^. Determination of *h* differentiated the color perceived in the fruits, with a tendency of red to yellow. In this work, CIELAB (L*, a*, b*) and CIELCH (L*, C* and *h*) values explained the differences in pericarp color of the fruits obtained from the different treatments [62].

Currently, there are no reports of the evaluation of color in fruits obtained from plants treated with metal NPs. However, in *Capsicum*, the color of the fruit is determined mainly by the composition and concentration of carotenoids [63]. The biosynthesis of these compounds and their accumulation in fruits is influenced by different factors as a defense mechanism against various biotic and abiotic stresses [64,65].

García-Gómez et al. [21] evaluated the effect of ZnO NPs and ZnSO_4_ on antioxidant defenses in tomato plants, the results indicated that ZnO NPs generated greater toxicity and stimulated the concentration of carotenoids and biological markers of oxidative stress (ROS). Some studies, have shown that ZnO NPs can induce oxidative stress and modify the activity of antioxidant enzymes and non-enzymatic antioxidant compounds [19,66], which operate together to protect plant cells against oxidative damage. Pérez-Labrada et al. [67] reported that foliar application of Cu NPs induced an increase in the content of vitamin C and carotenoids (lycopene) in fruits compared to the levels obtained in the control treatment. This study the evaluation of plant growth showed a greater sensitivity to the application of ZnO NPs, since at 1000 mg L^−1^ stimulated the growth of plants, while at 2000 mg L^−1^ there was a reduction in the development of the plants possibly due to a phyto-toxic effect. However, the fruits obtained in both treatments reported the greatest increases in nutraceutical and functional compounds, the increases observed in the accumulation of these compounds reflect a redox state modified by the application of ZnO NPs that affect the level of cellular ROS [68].

Therefore, these results suggest that the fruits of the plants treated with the foliar application of ZnO NPs intensified their orange color due to the increased production of carotenoids in response to oxidative stress. The main function of carotenoids is the protection of cells and organelles against oxidative damage, which they achieve by interacting with singlet oxygen molecules (O_2_), eliminating peroxyl radicals (LOO·) and preventing accumulation [69]. Although the investigation of the effect of the application of ZnO NPs on the chromatic characteristics of the fruit, the accumulation of carotenoids and phenolic compounds is very scarce, the role of the previous compounds against the stress induced by metallic NPs is uncertain [68].

### 3.5. Quality of Habanero Pepper Fruit

The results in Table 3 show that foliar application of ZnO NPs and ZnSO_4_ significantly affected (*p* ≤ 0.01) titratable acidity of fruits. Maximum titratable acidity (0.155 ± 0.0055) was obtained with 2000 mg L^−1^ of ZnO NPs, while the minimum value (0.119 ± 0.0050) of titratable acidity was recorded in fruits from the control treatment. In addition, fruit pH was also affected (*p* ≤ 0.01) by the treatments with ZnO NPs and ZnSO_4_ (Table 3). The highest value was obtained with the control treatment (5.63 ± 0.114), while the lowest pH (5.40 ± 0.040) was found with ZnO NPs at 2000 mg L^−1^, however, no significant differences were observed between the Zn-based treatments with different concentrations. Fruit pH correlates with acidity, and citric acid is the primary organic acid found in most fruits [70]. With respect to soluble solids, all treatments showed higher values than the control treatment. The treatment level with 2000 mg L^−1^ ZnO NPs produced the highest amount (11.33 ± 0.72), while the lowest value was obtained in the control (9.32 ± 0.79). As a result, fertilization with ZnO NPs and ZnSO_4_ also significantly increased (*p* ≤ 0.05) fruit firmness. The highest increase (15.35 ± 0.54) was obtained with 2000 mg L^−1^ of ZnO NPs, and the lowest value was found (14.79 ± 0.63) in fruits from the control treatment.

Our results are in agreement with those obtained by Davarpanah et al. [1], who mentioned that foliar fertilization with ZnO NPs at a concentration of 120 mg L^−1^ led to significant improvements in quality of pomegranate fruits, including increases of 4.4–7.6% in soluble solids, decreases of 9.5–29.1% in titratable acidity, increases of 20.6–46.1% in maturity index, and increments of 0.28–0.62% in juice pH. The physical characteristics of the fruit were not affected. Previous studies have reported that foliar application of micronutrients such as Zn and Fe are essential to increase yield, quality, and content of ascorbic acid in tomato fruits [71].

In this study, the increases in soluble solids and firmness of fruits under fertilization with ZnO NPs and ZnSO_4_ could be attributed to the role that Zn plays in the synthesis and transference of carbohydrates and proteins [72], in addition to maintaining the structural stability of cell membranes [73]. These facts indicate that Zn availability and concentration by foliar fertilization with ZnO NPs during the main stages of vegetative growth were more effective and have important physiological functions that could improve fruit quality. These results might be attributed to Zn movement from leaf tissues through the phloem to fruits at the moment of development and maturation [23].

### 3.6. Capsaicin and Dihydrocapsaicin Content

**Figure 3:**
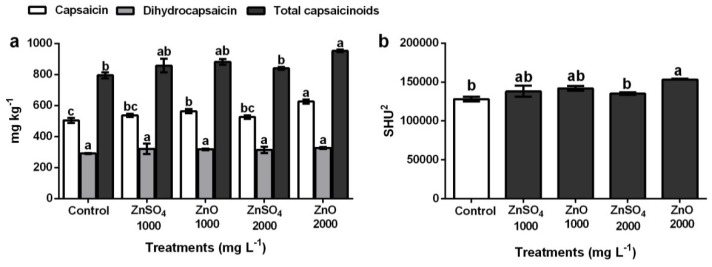
Capsaicinoids content in habanero pepper fruits from plants treated with ZnO NPs and ZnSO_4_. (**a**) capsaicin, dihydrocapsaicin and total capsaicinoids, (**b**) Scoville Heat Units (SHU^2^). Values are the average of three repetitions. Means (*n* = 3). Bars represent the standard deviation of the mean. Different letters in each bar mean that the treatments were statistically different (Tukey, *p* ≤ 0.05).

**Figure 4:**
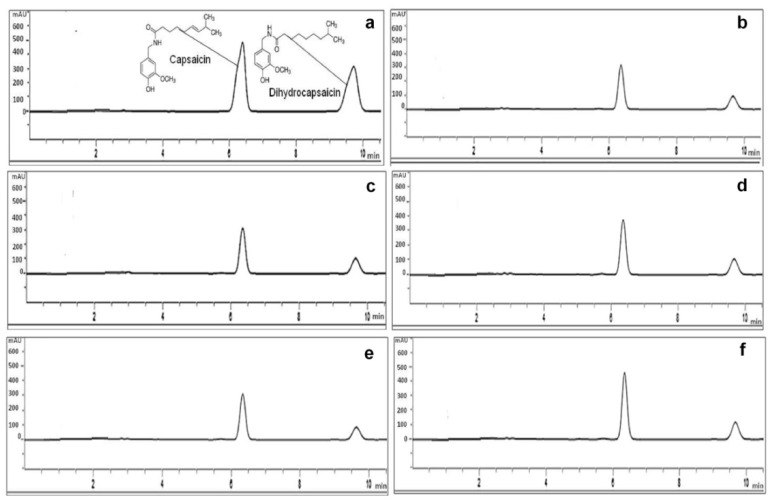
Chromatograms for capsaicin and dihydrocapsaicin in habanero pepper fruits from plants treated with ZnO NPs and ZnSO_4_, (**a**) standards, (**b**) control, (**c**) 1000 mg L^−1^ ZnSO_4_, (**d**) 1000 mg L^−1^ ZnO NPs, (**e**) 2000 mg L^−1^ ZnSO^4^, (**f**) 2000 mg L^−1^ ZnO NPs.

Results indicated that capsaicin content was significantly affected (*p* ≤ 0.01) by application of foliar fertilization with ZnO NPs and ZnSO_4_ (Figure 3a). The highest accumulation was detected at 2000 mg L^−1^ of ZnO NPs (625.44 ± 14.47 mg kg^−1^), which was 19.3% higher than the control treatment (504.60 ± 16.73 mg kg^−1^). The same trend was observed in dihydrocapsaicin content: although the treatments were statistically equal (*p* ≤ 0.423), accumulation increased in both treatments (Figure 3a). Treatment at 2000 mg L^−1^ of ZnO NPs caused the greatest increase with 10.9% (326.71 ± 6.50 mg kg^−1^) compared to the control (290.84 ± 2.80 mg kg^−1^). Quantitative analysis of capsaicinoids by HPLC revealed that the total content of these compounds increased in fruits obtained from plants treated with ZnO NPs (Figure 3a). The highest concentration of capsaicinoids was detected in mature fruits from plants treated with 2000 mg L^−1^ of ZnO NPs, with a maximum value of 952.15 ± 8.17 mg kg^−1^ that exceeded by 16.4% the control (795.43 ± 19.54 mg kg^−1^), while the values obtained with ZnSO_4_ at 2000 mg L^−1^ were statistically the same as the control. The chromatogram in Figure 4a corresponds to capsaicin and dihydrocapsaicin standards, with retention times of 6.354 min (peak 1) and 9.661 min (peak 2), respectively.

There is a direct correlation between total capsaicinoids content and pungency in pepper fruits. Fruit SHU also increased significantly (*p* ≤ 0.01) by fertilization with ZnO NPs (Figure 3b). The highest increase of 16.4% (153,296.1 ± 1315.79 SHU^2^) was obtained at a concentration of 2000 mg L^−1^ with ZnO NPs, while the control had the lowest accumulation (128,065.0 ± 3146.93 SHU^2^). On the other hand, the values obtained for SHU^2^ with ZnSO_4_ at 2000 mg L^−1^ did not show significant increases. The SHU scale measures the pungency of peppers, and it depends on the concentration of capsaicin and dihydrocapsaicin. The SHU can be classified as (1) non-spicy (0–700 SHU^2^), (2) slightly spicy (700–3000 SHU^2^), (3) moderately spicy (3000–25,000 SHU^2^), (4) highly spicy (25,000–70,000 SHU^2^), and (5) very hot (>80,000 SHU^2^) [74]. Based on our results, the habanero pepper fruits harvested in this study are classified as very spicy fruits; however, total capsaicinoid concentration, and thus SHU^2^, increased sharply with the application of ZnO NPs at high concentrations (2000 mg L^−1^).

This behavior relates to capsaicinoids as main antioxidants in peppers [35] and their protective functions against ROS [75]. Therefore, it is possible that the sharp increase observed is due to oxidative stress caused by ZnO NPs [19], because metal NPs alter Ca^2+^ and ROS concentrations involved in cell signaling and complex physiological and biochemical changes at the organism level [76]. In this case, the plant defense system accumulated higher concentrations of enzymatic and non-enzymatic antioxidant compounds, which resulted in greater capsaicinoid accumulation (Figure 3). Figure 4b,c,e show the chromatograms and retention times for the compounds of the control fruits and the ZnSO_4_ treatments, while Figure 4d,f represent the retention times obtained for the treatments with ZnO NPs.

### 3.7. Total Phenols, Total Flavonoids and Condensed Tannins

Results indicate that the foliar application of ZnO NPs significantly affected (*p* ≤ 0.01) phenols and total flavonoids (soluble + bound) content in the fruits. In all analyses of phenolic compounds, the soluble portion had the highest concentrations of phenols and flavonoids, while the lowest concentration was found in bound compounds. The highest concentration of total phenols (soluble + bound) was obtained with applications of ZnO NPs: in the treatment with 1000 mg L^−1^ the content was 1442.76 mgGAE kg^−1^, while for 2000 mg L^−1^ it was 1504.60 mgGAE kg^−1^ (Table 4), exceeding by 10.8% and 14.5% the control treatment. The concentrations obtained with ZnSO_4_ were 1290.55 mgGAE kg^−1^ for 1000 mg L^−1^ and 1299.93 mgGAE kg^−1^ for 2000 mg L^−1^, without presenting significant statistical differences in comparison with the control. When compared to values obtained with ZnO NPs at the same concentrations, these amounts were 10.5% and 13.6% smaller respectively.

This behavior was also observed for total flavonoids content (Table 4). The highest concentration of total flavonoids (soluble + bound) was found at 1000 mg L^−1^ and 2000 mg L^−1^ of ZnO NPs, with values of 237.17 mgCatE kg^−1^ and 251.50 mgCatE kg^−1^, respectively, exceeding by 22.5% and 26.9% the control treatment. However, concentrations obtained with ZnSO_4_ at 1000 mg L^−1^ (196.24 mgCatE kg^−1^) and 2000 mg L^−1^ (196.35 mgCatE kg^−1^) were 21.9% and 17.2% lower than those obtained at the same concentrations of ZnO NPs and when compared with the control, there are no significant statistical differences. The content of phenolic compounds is considered one of the most important nutraceutical value parameters in habanero pepper fruits [77]. Similar concentrations of phenolic compounds have been found in orange habanero peppers (169.97 mg GAE 100 g^−1^), while other genotypes of habanero peppers grown in Yucatan, Mexico have lower concentrations (20.54 mg 100 g^−1^ to 20.75 mg 100 g^−1^) [78]. The presence of condensed tannins was not detected in any of the samples. In this study, the effects caused (beneficial and toxic) by the foliar application of ZnO NPs in plants were higher than those caused by ZnSO_4_ in the same concentrations. For example, the highest accumulation of phenolic compounds and total flavonoids was obtained in the harvested fruits of the plants that were treated with 1000 and 2000 mg L^−1^ ZnO NPs. To date, there are no reports comparing the effect of ZnO NPs and ZnSO_4_ on the accumulation of bioactive compounds in habanero pepper fruits. However, our findings suggest that the effect could be related to a specific mechanism of the nanoparticles in the plant system, since the ZnO NPs treatments generated the greatest physiological and biochemical changes in the plants. Some authors mentioned that the toxicity generated by the ZnO NPs is due to the internalization of the NPs, the accumulation in the tissue and the dissolution of the zinc ions [79], this suggests that the ZnO NPs can induce toxicity through the activity of the ions that are released during a prolonged period and can generate a greater stimulation in the formation of ROS.

Additionally, the difference in accumulation of phenolic compounds caused by ZnO NPs differs from those caused by ZnSO_4_ in the same concentrations (1000 mg L^−1^ and 2000 mg L^−1^), this can be explained because ZnSO_4_ is highly soluble and when applied to the leaves of plants it can fall rapidly, therefore, the bioavailability of Zn ions with the use of ZnSO_4_ during an extended period is not safe [9,16]. Therefore, it is important to take into account that the foliar application of ZnSO_4_ could not generate the same stress as the ions obtained by the ZnO NPs, since the NPs have a higher transport potential and, therefore, a greater bioavailability and absorption that allows them to interact with intracellular structures that stimulate ROS formation [80,81]. However, other authors emphasized that the phytotoxicity of ZnO NPs cannot be explained only by dissolved ions since the properties of NPs can be affected by the means of exposure to plants [82,83].

Zafar et al. [84] indicated that the application of ZnO NPs induced oxidative stress in the shoots of *Brassica nigra* and, interestingly, observed an increase in the non-enzymatic antioxidant molecules such as phenolic compounds and flavonoids due to the accumulation of ROS. The results of the experiment conducted by Pinedo-Guerrero et al. [35] also reported that the application of Cu NPs in chitosan-PVA hydrogels at a concentration of 2.0 mg in jalapeño pepper plants increased the accumulation of phenols in fruits (64.71 mgGAE 100 g^−1^), surpassing in 5.9% the control, the increase was associated with an increase in oxidative stress generated by ROS.

Plants have developed various protection mechanisms to limit oxidative damage caused by ROS through the production of antioxidants such as phenols, carotenoids and antioxidant enzymes [26]. Phenolic compounds play a prominent role in detoxification mechanisms of ROS [85] as electron donors in organelle structures and can directly eliminate the molecular species of active oxygen, mainly due to their redox properties. They act in absorption and neutralization of free radicals, extinction of singlet and triplet oxygen or decomposition of peroxides [86]. This behavior explains the highest accumulation of phenols and flavonoids in the fruits from plants exposed to treatments with ZnO NPs.

### 3.8. Antioxidant Capacity

In Table 5, significant differences (*p* ≤ 0.01) were observed among treatments in the antioxidant capacity assays of ABTS, DPPH, and FRAP. In all analyses, the soluble portion had the highest antioxidant capacity. Results indicate that total antioxidant activity (soluble + bound) was from 83.28 mmolTE kg^−1^ to 98.49 mmolTE kg^−1^, from 145.29 mmolTE kg^−1^ to 213.16 mmolTE kg^−1^, and from 235.25 mmolTE kg^−1^ to 296.06 mmolTE kg^−1^ in ABTS, DPPH, and FRAP, respectively (Table 5). According to results of phenolic content, the three methods of antioxidant capacity had the highest values for treatments with ZnO NPs at concentrations of 1000 mg L^−1^ and 2000 mg L^−1^ and were statistically different from the control and ZnSO_4_ treatments (Table 5). Correlations between total phenolic content (r = 0.95, 0.94, and r = 0.80, respectively), total flavonoids (r = 0.95, 0.89, and r = 0.80, respectively) and antioxidant capacity measured by FRAP, DPPH, and ABTS suggest that higher values of phenolic compounds in the habanero peppers are related to greater antioxidant capacity. Similar patterns have been reported in jalapeño pepper fruits [35], peppers of the *Capsicum* genus [87] and jalapeño and serrano peppers [88].

Previous studies have shown that ZnO NPs can generate cytotoxicity due to the production of ROS [55]. The induction and biosynthesis of phenolic compounds are related to stress caused by heavy metals. This response may explain the large accumulation of these compounds in fruits from plants exposed to ZnO NPs [89], and it could be the main reason for the observed higher antioxidant activities [90]. A study conducted by Zare et al. [91] showed that synthesized ZnO NPs had higher antioxidant activity (ABTS and DPPH) compared to ZnO microparticles. The biological and antioxidant effectiveness of ZnO NPs depends on particle size and shape [12]. In this study, the application of ZnO NPs generated the highest accumulation of phenolic compounds in fruits and resulted in increases in antioxidant activities. 

## 4. Conclusions

The application of ZnO NPs affected the development of pepper plants. At a concentration of 1000 mg L^−1^, it promoted plant growth, and increased number and average weight of the fruits, while at 2000 mg L^−1^, it promoted negative effects on growth and development of the crop. Therefore, the ZnO NPs effect depends on the concentration applied. In contrast, the effect of the application of ZnSO_4_ at concentrations of 1000 and 2000 mg L^−1^ was lower and had minor effects on crop yield. On the other hand, foliar application of ZnO NPs at 2000 mg L^−1^ led to significant improvements in fruit quality, including increases in titratable acidity, soluble solids, and decreases in pH without significant differences between treatments with Zn. Likewise, accumulation of total capsaicinoids in fruits from plants treated with 1000 mg L^−1^ and 2000 mg L^−1^ of ZnO NPs reached 881.57 mg Kg^−1^ and 952.15 mg Kg^−1^, respectively, which resulted in significant increases in SHU. In the same way, application of ZnO NPs at 1000 mg L^−1^ and 2000 mg L^−1^ increased phenols and total flavonoids (soluble + bound) content in habanero pepper fruits that resulted in increased antioxidant capacity according to ABTS, DPPH, and FRAP assays.

In summary, results of this study demonstrate the influence of the foliar application of ZnO NPs and ZnSO_4_ on development and yield of habanero pepper plants.

## Figures and Tables

**Table 1:** Effect of foliar application of ZnO NPs and ZnSO_4_ on fruit development and biomass accumulation.

Treatments (mg L^−1^)	Number of Fruits	Average Fruit Weight (g)	Total Weight of Fruits (g)	Fresh Aerial Biomass (g)	Dry Aerial Biomass (g)
Control	54.20 ± 2.28 c	8.80 ± 0.22 ab	476.97 ± 23.74 ab	893.85 ± 11.00 b	294.80 ± 11.33 c
ZnSO_4_ 1000	58.50 ± 1.29 b	9.12 ± 0.11 ab	533.78 ± 11.93 ab	905.09 ± 5.88 b	310.81 ± 3.44 b
ZnO NPs 1000	64.00 ± 2.24 a	9.46 ± 0.12 a	605.30 ± 17.27 a	925.64 ± 4.09 a	324.91 ± 5.09 a
ZnSO_4_ 2000	50.25 ± 1.50 d	8.46 ± 0.07 b	425.20 ± 11.19 b	879.46 ± 5.99 c	278.56 ± 8.59 d
ZnO NPs 2000	46.80 ± 2.39 d	8.29 ± 0.11 b	387.99 ± 18.71 b	868.22 ± 2.42 c	264.42 ± 4.92 e

Values are the average of five repetitions. Means (*n* = 5) ± standard deviation. Different letters in each column means that the treatments were statistically different (Tukey, *p* ≤ 0.05).

**Table 2:** Chromatic characteristics in habanero pepper fruits obtained from plants treated with ZnO NPs and ZnSO_4_.

Treatments (mg L^−1^)	Chromatic Parameter					
	L*	a*	b*	C*	*h*	View
Control	50.02 ± 1.08 b	30.57 ± 1.93 a	38.92 ± 1.33 b	49.37 ± 1.97 b	51.77 ± 1.46 a	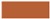
ZnSO_4_ 1000	51.29 ± 1.76 b	32.06 ± 0.85 a	41.98 ± 1.11 a	50.77 ± 1.32 b	52.33 ± 1.00 a	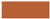
ZnO NPs 1000	51.99 ± 1.11 ab	32.33 ± 1.45 a	42.48 ± 2.13 a	51.81 ± 0.76 ab	53.71 ± 1.24 a	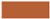
ZnSO_4_ 2000	51.92 ± 1.46 ab	32.07 ± 0.84 a	41.91 ± 1.42 a	51.75 ± 1.01 ab	53.30 ± 1.25 a	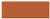
ZnO NPs 2000	53.46 ± 1.15 a	33.45 ± 1.20 a	43.59 ± 1.47 a	53.43 ± 1.64 a	53.84 ± 1.44 a	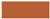

Values are the average of five repetitions. Means (*n* = 5) ± standard deviation. Different letters in each column means that the treatments were statistically different (Tukey, *p* ≤ 0.05).

**Table 3:** Effect of foliar application of ZnO NPs and ZnSO_4_ on fruit development and biomass accumulation.

Treatments (mg L^−1^)	TA (%)	pH	Soluble Solids (%)	Firmness (N)	Cutting Force (N)
Control	0.119 ± 0.0050 d	5.63 ± 0.114 a	9.32 ± 0.79 d	14.79 ± 0.63 b	11.35 ± 0.83 a
ZnSO_4_ 1000	0.126 ± 0.0012 cd	5.49 ± 0.030 b	10.18 ± 0.38 c	14.94 ± 0.65 ab	11.39 ± 0.77 a
ZnO NPs 1000	0.134 ± 0.0025 b	5.42 ± 0.048 b	10.77 ± 0.60 b	15.11 ± 0.68 ab	11.43 ± 0.64 a
ZnSO_4_ 2000	0.130 ± 0.0020 bc	5.50 ± 0.025 b	10.48 ± 0.52 bc	15.20 ± 0.63 ab	11.36 ± 0.72 a
ZnO NPs 2000	0.155 ± 0.0055 a	5.40 ± 0.040 b	11.33 ± 0.72 a	15.35 ± 0.54 a	11.32 ± 0.47 a

Values are the average of five repetitions. Means (*n* = 5) ± standard deviation. Different letters in each column means that the treatments were statistically different (Tukey, *p* ≤ 0.05).

**Table 4:** Content of total phenols and total flavonoids (soluble + bound) in habanero pepper fruits obtained from plants treated with ZnO NPs and ZnSO_4_.

Treatments (mg L^−1^)	Total Phenolics (mgGAE kg^−1^)			Total Flavonoids (mgCatE kg^−1^)		
	Free	Bound	Total	Free	Bound	Total
Control	1154.85 ± 10.55 b	113.50 ± 4.30 b	1286.35 b	114.35 ± 5.52 b	69.30 ± 2.71 c	183.65 b
ZnSO_4_ 1000	1168.44 ± 17.76 b	122.11 ± 6.55 b	1290.55 b	116.95 ± 10.22 b	79.28 ± 5.83 bc	196.24 b
ZnO NPs 1000	1293.42 ± 28.30 a	149.34 ± 6.45 a	1442.76 a	144.63 ± 9.53 a	92.54 ± 5.39 ab	237.17 a
ZnSO_4_ 2000	1176.24 ± 15.61 b	123.68 ± 4.98 b	1299.93 b	119.73 ± 6.89 a	76.62 ± 4.43 c	196.35 b
ZnO NPs 2000	1347.41 ± 30.06 a	157.18 ± 7.58 a	1504.60 a	155.01 ± 8.04 a	96.50 ± 6.20 a	251.50 a

Values are the average of three repetitions. Means (*n* = 3) ± standard deviation. Different letters in each column means that the treatments were statistically different (Tukey, *p* ≤ 0.05).

**Table 5:** Antioxidant activity in habanero pepper fruits obtained from plants treated with ZnO NPs and ZnSO_4._

Treatments (mg L^−1^)	ABTS (mmolTE kg^−1^)		DPPH (mmolTE kg^−1^)		FRAP (mmolTE kg^−1^)	
	Free	Bound	Free	Bound	Free	Bound
Control	76.60 ± 3.19 b	6.68. ± 4.30 c	112.97 ± 5.52 b	32.32 ± 2.71 c	172.75 ± 4.68 b	61.50 ± 3.25 b
ZnSO_4_ 1000	83.33 ± 3.18 ab	7.95 ± 6.55 b	121.67 ± 10.22 b	35.60 ± 5.83 c	180.86 ± 5.19 b	65.19 ± 3.51 b
ZnO NPs 1000	86.77 ± 2.75 a	8.75 ± 6.45 ab	154.98 ± 9.53 a	49.70 ± 5.39 b	198.22 ± 5.14 a	80.51 ± 0.66 a
ZnSO_4_ 2000	81.08 ± 3.18 ab	8.12 ± 4.98 b	130.31 ± 6.89 b	38.19 ± 4.43 c	180.30 ± 6.73 b	67.61 ± 3.08 b
ZnO NPs 2000	89.14 ± 2.96 a	9.35 ± 7.58 a	156.55 ± 8.04 a	56.61 ± 6.20 a	212.21 ± 8.35 a	83.85 ± 1.97 a

Values are the average of five repetitions. Means (*n* = 3) ± standard deviation. Different letters in each column means that the treatments were statistically different (Tukey, *p* ≤ 0.05).
